# Machine learning based urban sprawl assessment using integrated multi-hazard and environmental-economic impact

**DOI:** 10.1038/s41598-024-62001-6

**Published:** 2024-06-11

**Authors:** Anjar Dimara Sakti, Albertus Deliar, Dyah Rezqy Hafidzah, Adria Viola Chintia, Tania Septi Anggraini, Kalingga Titon Nur Ihsan, Riantini Virtriana, Deni Suwardhi, Agung Budi Harto, Sella Lestari Nurmaulia, Adiwan Fahlan Aritenang, Akhmad Riqqi, Andri Hernandi, Budhy Soeksmantono, Ketut Wikantika

**Affiliations:** 1 Geographic Information Sciences and Technology Research Group, Faculty of Earth Sciences and Technology, Institut Teknologi Bandung, Bandung, 40132 Indonesia; 2 Center for Remote Sensing, Institut Teknologi Bandung, Bandung, 40132 Indonesia; 3 Center for Spatial Data Infrastructure, Institut Teknologi Bandung, Bandung, 40132 Indonesia; 4 Spatial System and Cadastre Research Group, Faculty of Earth Sciences and Technology, Institut Teknologi Bandung, Bandung, 40132 Indonesia; 5 Department of Urban and Regional Planning, School of Architecture, Planning and Policy Development, Institut Teknologi Bandung, Bandung, 40132 Indonesia

**Keywords:** Environmental impact, Natural hazards, Scientific data

## Abstract

The increasing demand for land development due to human activities has fueled urbanization. However, uncontrolled urban development in some regions has resulted in urban environmental problems arising from an imbalance between supply and demand. This study aims to develop an integrated model for evaluating and prioritizing the management of hazardous urban sprawl in the Bandung metropolitan region of Indonesia. The novelty of this study lies in its pioneering application of long-term remote sensing data-based and machine learning techniques to formulate an urban sprawl priority index. This index is unique in its consideration of the impacts stemming from human economic activity, environmental degradation, and multi-disaster levels as integral components. The analysis of hazardous urban sprawl across three distinct time periods (1985–1993, 1993–2008, and 2008–2018) revealed that the 1993–2008 period had the highest increase in human economic activity, reaching 172,776 ha. The 1985–1993 period experienced the highest level of environmental degradation in the study area. Meanwhile, the 1993–2008 period showed the highest concentration of multi-hazard locations. The combined model of hazardous urban sprawl, incorporating the three parameters, indicated that the highest priority for intervention was on the outskirts of urban areas, specifically in West Bandung Regency, Cimahi, Bandung Regency, and East Bandung Regency. Regions with high-priority indices require greater attention from the government to mitigate the negative impacts of hazardous urban sprawl. This model, driven by the urban sprawl priority index, is envisioned to regulate urban movement in a more sustainable manner. Through the efficient monitoring of urban environments, the study seeks to guarantee the preservation of valuable natural resources while promoting sustainable urban development practices.

## Introduction

The high demand for land development caused by human activities has stimulated economic growth, infrastructure development, and urban population growth^1,2^. Rapid urbanization has occurred in recent decades, with the proportion of the population living in urban areas increasing from 30% in 1950 to 55% in 2018 and projected to reach 68% by 2050^3^. Liu et al.^4^ mapped the global annual urban dynamics from 1985 to 2015 and found that the global urban area increased by 9687 km^2^ per annum. This has resulted in the depletion of natural resources, food shortages, land degradation, and biodiversity loss, all of which have an impact on community quality^5^. Uncontrolled urban development can give rise to various environmental problems, leading to potential dangers such as changes in groundwater levels and conditions, increased emissions, waste production, and wastewater generation^6–8^, as well as land cover changes that contribute to elevated atmospheric carbon^9^. Additionally, urban development is associated with climate change and the urban heat island effect, both of which influence energy consumption^10,11^. Efforts to manage the expansion of urban areas continue by developing studies and planning strategies that can be utilized by policymakers^12^. During the development of studies, there are several parameters that need to be considered for identifying urban sprawl areas, which include biodiversity, health, land use, and socio-economic factors^13–15^. In addition, the disaster exposure parameter requires attention, given that some cities are susceptible to economic losses and casualties resulting from multi-hazards in major urban areas^16^.

Several studies have specifically utilized geospatial technologies, such as spatial analysis and remote sensing, to monitor and assess urban expansion. Behnisch et al.^17^ presented a global perspective on the rapid expansion of urban sprawl, distinguishing hotspots and trends that have occurred since 1990. Boori et al.^18^ employed remote sensing and GIS to monitor and model urban sprawl dynamics in Kuala Lumpur, Malaysia, within a specific urban context. He et al.^19^ demonstrated a technological approach by utilizing a fully convolutional network to detect global urban expansion trends that have occurred over the past three decades. Li and Jiang^20^ redirected their focus towards the interplay between urban development and natural ecosystems, investigating how urbanization-induced disturbances affect the structure, functions, and biodiversity of forests. Shawly^21^ adopted a pragmatic approach by evaluating the implementation of the Compact City Model in Jeddah as a tool to control urban sprawl in a sustainable manner. Collectively, these studies, which have specifically utilized geospatial technologies, present a diverse array of approaches, ranging from global analyses to localized case studies. As such, they provide multifaceted insights into the complexities of urban sprawl.

Expanding upon the exploration of urban sprawl, several other studies have further enriched our understanding of this phenomenon. Gennaio et al.^22^ evaluated the effectiveness of urban growth boundaries utilizing a land use plan. Another study conducted by Jiao et al.^23^ employed an elasticity coefficient approach to assess urban expansion. Cai et al.^24^ analyzed the coordination between urban expansion, population growth, and economic development using a decoupling model. Abedini et al.^25^ assessed urban sprawl phenomena using landscape metrics and the black-and-white hypothesis. Moreover, a recent study by Cai et al.^26^ analyzed the rationality of emerging megacity urban expansion based on spatiotemporal characteristics. Although these studies have generated complex frameworks by integrating various data analyses, their primary focus has predominantly revolved around socio-economic issues and land changes. The potential of incorporating long-term remote sensing data in conjunction with machine learning approaches to detect urban sprawl phenomena has been acknowledged but not fully realized, resulting in unexplored prospects for future research and advancements in the field.

The Bandung metropolitan area in Indonesia has witnessed a remarkable transformation, with its population increasing threefold over a span of 35 years since 1970. This has resulted in the substantial expansion of built-up areas and a notable increase in land and building ownership^27^. However, this unrestrained urban growth has given rise to a plethora of issues, primarily stemming from an imbalance between the supply and demand of resources. As a response, the West Java government has enacted regulations addressing building proliferation, which include restrictions on elevation and slope in specific areas^28^. As response to these challenges, Pravitasari et al.^29^ focused on identifying the driving forces of urban expansion and their environmental impact in the Jakarta-Bandung Mega Urban Region. Fuadina et al.^30^ analyzed the factors influencing urban sprawl, specifically in the Bandung Basin Area. Margono et al.^31^ investigated the impact of housing transformation on the quality of life in the peri-urban area of North Bandung. Each of these studies provides a unique perspective, collectively enhancing our full comprehension of urban development challenges in the Bandung metropolitan area.

Expanding on the advanced groundwork, this study aims to develop an urban sprawl priority index in order to identify high-priority areas for intervention and mitigation in the Bandung metropolitan area. The analysis is utilized to evaluate the sustainability of urbanization using three main parameters: human economic activity impact, environmental degradation, and multi-disaster. To achieve this objective, there are several supporting goals: the establishment of a human-economy activity impact model using long-term population density and nighttime light data; the development of an environmental degradation model through the integration of surface temperature increases and long-term air pollution data; and the modeling of multi-disaster events, specifically floods and landslides, using machine learning approaches. The novelty of this study lies in its pioneering application of long-term remote sensing data-based machine learning techniques to formulate an urban sprawl priority index. This index is unique in its consideration of the impacts stemming from human economic activity, environmental degradation, and multi-disaster levels as integral components. The application of machine learning in this context offers distinct advantages, enhancing the effectiveness and efficiency of data processing. The anticipated outcome is the development of an integrated urbanization evaluation model. The purpose of this model, guided by the urban sprawl priority index, is to effectively regulate urban development in a sustainable manner. Through the efficient oversight of urban environments, the study aims to ensure the preservation of valuable natural resources and encourage sustainable urban development practices.

## Methods

### Study area

**Figure 1:**
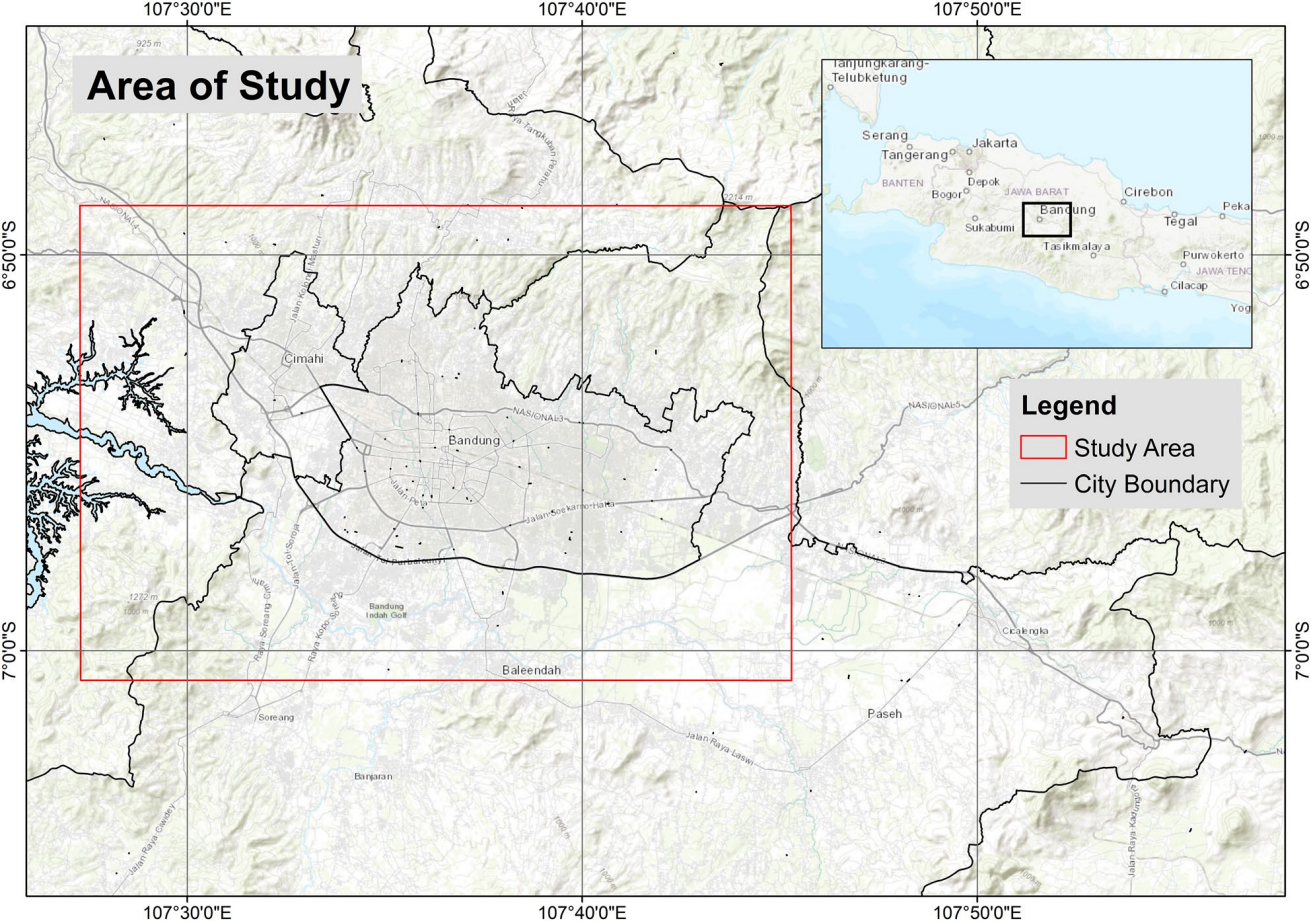
Study area of Bandung metropolitan area, West Java Province, Indonesia. The Bandung metropolitan region consists of several administrative areas, including Bandung City, Cimahi City, and parts of Bandung Regency, West Bandung Regency, and Sumedang Regency.

The study area comprises the Bandung metropolitan or Bandung Raya region (Fig. 1), which consists of Bandung City, Cimahi City, Bandung Regency, West Bandung Regency, and parts of Sumedang Regency, with a total area of approximately 3500 km^2^ and a population of 8.6 million people in 2021^32^. High economic growth and urbanization in the Bandung Raya region pose formidable challenges in achieving sustainable urban development while maintaining a high quality of life. Geographically, this region is located between 6° 51′ 36″ and 6° 55′ 8″ S and 107° 33′ 18″ and 107° 34′ 50″ E, with an elevation ranging from 675 to 1050 m above sea level.

### Data used in this study

This study attempted to integrate multiple geospatial data products, both vector and raster. On the basis of their application, the data used in this study could be categorized into four main phases: the human economic activity modeling phase, the environmental degradation modeling phase, the multi-hazard modeling phase, and the integration phase for developing the hazardous urban sprawl index. Table 1 presents the types and characteristics of the data used in this study. The analysis of annual urban movement in this study utilized global impervious surface data with a resolution of 30 m^33^, covering the period from 1985 to 2018. The parameters used in the human economic activity phase included nighttime light and population data. Satellite nighttime light imagery has been applied in various studies relating to socio-economic aspects, in which it represented urban infrastructure development^34,35^. The Corrected Visible Infrared Imaging Radiometer Suite (VIIRS) Day/Night Band Composites was used, containing monthly data and a raster resolution of 463.83 m^36^, in this study, the mean annual data for 2020 was processed. Population density data was obtained from the WorldPop Global Project Population Data with a raster resolution of 92.77 m^37^. This dataset provides estimates of the population residing in each grid cell, and the average population values for 2020 were used in this study.

**Table 1:** Characteristics of the data used in the study.

Data	Product	Provider	Type	Year	Reference
Impervious surface	Tsinghua FROM-GLC Year of Change to Impervious Surface	Tsinghua University	Raster 30 m	1985–2018	^33^
Night light	VIIRS Nighttime Day/Night V1	CSM	Raster 500 m	Monthly 2012–2022	^36^
Population	WorldPop Global Project Population	WorldPop	Raster 100 m	Annual 2000–2020	^37^
Aerosol optical depth	MCD19A2.006:	NASA LP DAAC	Raster 1000 m	Daily 2000–2022	^38^
Land surface temperature	MOD11A1.061 Terra Land Surface Temperature	NASA LP DAAC	Raster 1000 m	Daily 2000–2022	^39^
Land cover	ESA LC	ESA	Raster 10 m	2020	^40^
NDVI	Landsat 8 Tier 1 TOA Reflectance	USGS	Raster 30 m	Mean 2021–2022	^41^
DEM	DEMNAS	BIG	Raster 10 m	2008	^42^
Precipitation	CHIRPS DAILY	UCSB	Raster 10 km	Mean 2021–2022	^43^
Landslide hazard point	BNPB, Landslide Global Catalog	BNPB, NASA	Vector Point	2010–2020	^44–46^
Flood hazard point	BNPB, Global Flood Database	BNPB, DFO	Vector Point	2010–2020	
Residential density	Regional Spatial Plan of Bandung City	Bandung Gov	Polygon	2015	^47^

During the development phase of the environmental degradation model, two main data sources were used: the air pollution index and land surface temperature. The long-term effects of urbanization on air pollution, especially in large cities, demonstrate a positive correlation between high urbanization levels and increased long-term PM2.5 levels^48^. Global aerosol observations from satellite remote sensing can substantially improve population exposure estimates to PM2.5 air pollution^49,50^. In this study, the Moderate Resolution Imaging Spectroradiometer (MODIS) Terra & Aqua Multi-angle Implementation of Atmospheric Correction Land Aerosol Optical Depth (AOD) daily product was used as a representation of air quality analysis with a resolution of 1000 m^38^. In addition to air pollution, urban development affects land surface temperatures. The urban heat island effect is known to be substantially exacerbated by a land cover dominated by buildings^51^. This study utilizes the MODIS Terra Land Surface Temperature and Emissivity Daily Global dataset with a resolution of 1000 m to analyze land surface temperatures^39^.

**Figure 2:**
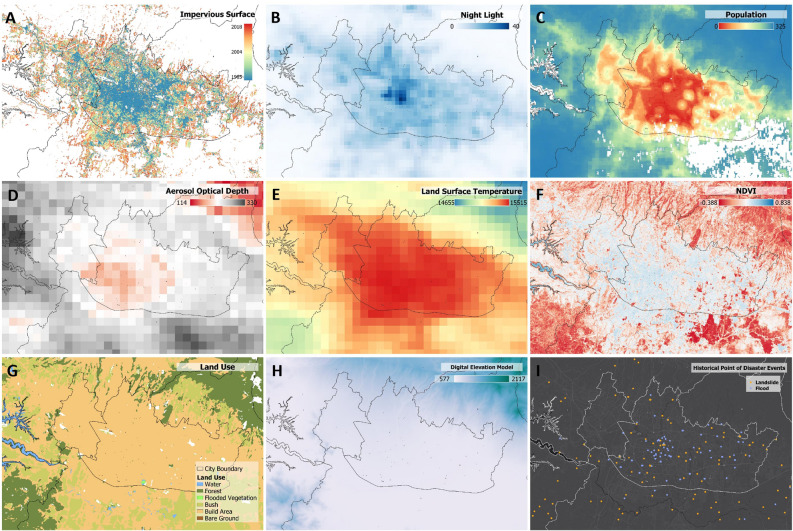
Visualization of the data used in the study: (**A**) Tsinghua FROM-GLC Year of Change to Impervious Surface, (**B**) VIIRS Nighttime Day/Night V1, (**C**) WorldPop Global Project Population, (**D**) MODIS Aerosol Optical Depth, (**E**) MODIS Land Surface Temperature, (**F**) LANDSAT Normalized Difference Vegetation Index (NDVI), (**G**) LANDSAT Land Use Land Cover, (**H**) ALOS Digital Elevation Model, (**I**) Multi-source historical events of floods and landslides in the Bandung Raya Region.

During the analysis phase of multi-hazard vulnerability, including flood and landslide occurrences, historical flood and landslide data were combined with land cover data, normalized difference vegetation index (NDVI) data, national digital elevation model (DEM) data, and precipitation data. The land cover data were derived from the European Space Agency (ESA) Worldcover product^40^, the NDVI data were obtained from Landsat 8 with a resolution of 30 m^41^, Indonesia National DEM data were obtained from Government official^42^ and the precipitation data were acquired from the Climate Hazards Group InfraRed Precipitation with Station data (CHIRPS) daily dataset^43^. Disaster inventories, which provide information on the locations of past disasters, include historical records of floods and landslides. The historical flood and landslide event data were obtained from various sources, including the Official data from national disaster management agency (BNPB)^44^, the global landslide catalog^45^, and the global flood database^46^. Figure 2 illustrates the visualization of the data used in this study. Historical data on floods in the Bandung area was used at 180 points, while landslides at 166 points were used as positive samples. Meanwhile, we randomly sampled non-hazard points in the flood-free and landslide-free zones. These served as negative samples. Ensuring a balance between positive and negative samples is crucial for achieving optimal model performance. The entire hazard dataset, comprising both positive and negative samples (i.e. presence and absence of floods or landslides), was divided into training and testing datasets. Inventory data collection was randomized and subsequently randomly divided into training (70%) and testing (30%) sets. The target class values (i.e. hazard points) were assigned a value of 1 if the sample indicated a positive occurrence of a disaster; conversely, if the sample did not indicate a disaster, the class value was set to 0. For the visualization of map data, we employed Quantum GIS (QGIS) software, version 3.36^52^.

### Machine learning linear regression

In this study, we conducted long-term analyses of human economic activity and environmental degradation using remote sensing satellite data. Linear regression machine learning models were employed to analyze the trends in nighttime light, population, AOD, and surface temperature data. The linear regression model aims to identify the best-fitting line that represents the linear relationship between predictor variables and the target variable. This process involves estimating regression coefficients that quantify the extent to which changes in the predictor variables impact the target variable^53^. The regression coefficients are obtained by comparing the trend of how the values of each parameter change over time using raster data^54^. The change analysis employs the linear regression method, as depicted in Eq. (1):

1 $${\text{y}}=\sum_{{\text{i}}=1}^{{\text{n}}}\frac{\sum_{{\text{i}}=1}^{{\text{n}}}({{\text{x}}}_{{\text{i}}}-\overline{{\text{x}} })({{\text{y}}}_{{\text{i}}}-\overline{{\text{y}} })}{{\left({{\text{x}}}_{{\text{i}}}-\overline{{\text{x}} }\right)}^{2}}{\text{x}}+\stackrel{-}{({\text{y}}}-{\text{a}}\overline{{\text{x}} }),$$

where y represents the dependent variable; × 1, × 2, …, xn refer to the independent variables that correspond to the time parameter; a denotes the rate of change, and b1, b2, …, bn signify the offset coefficients. A positive value for the rate of change indicates that the y variable experiences an annual increase. Conversely, a negative indicates that the y parameter decreases every year.

This analysis yields the rate of change in each data pixel, known as the relative rate of change (ROC). The ROC values were used to identify regions within the study area that had the highest increasing trends and the lowest decreasing trends. Positive linear regression coefficients indicated annual increasing trends, whereas negative coefficients indicated annual decreasing trends. Long-term remote sensing data trends were processed using the Google Earth Engine^55^, an open-source platform for big data remote sensing processing.

### Machine learning random forest classification

Random Forest (RF) is an ensemble learning method that constructs a large number of decision trees during the processing stage^56^. RF has been extensively utilized owing to its rapid processing speed and ability to generate classifications with lower error rates than those of other classification algorithms^57^. In a decision tree, the relationship between features and the target variable is represented by a series of conjunction conditions arranged in a tree-like structure from the top down^58^. Each variable must undergo all possible splits among the nodes of the tree. The minimum Gini value serves as the standard separation for a node, and the corresponding variable is considered the optimal variable. The Gini value can be evaluated using a specific threshold value. If the Gini value for a node in any subset is considerably lower than the threshold value, all samples are assigned to the same class without additional splitting. In contrast, subsets that exceed the threshold value are further divided into new subsets inside the tree. The Gini value, which quantifies the impurity level of each node, is calculated using the following formula:

2 $$Gini \left(t\right)=1-\sum_{j=1}^{k}{\left[p\left(j|t\right)\right]}^{2},$$

where $p\left(j|t\right)$ is the probability of class j at node t. When the value of $Gini \left(t\right)$ is 0, the sample data at node t is placed in the same class. When predicting the output of a decision tree, predetermined subsamples are randomly selected at each stage, and each output incorporates the values derived from the best voting^59^. Subsamples from the dataset are produced using the bootstrap resampling method^60^. Decision trees are recommended to address uncertainty issues because they can eliminate uncertainty and improve prediction accuracy^61^. In this study, RF analysis was employed in the multi-hazard analysis to assess landslide and flood hazards.

This study utilized the ROC curve as one of the approaches for determining the overall performance of the model, particularly in spatial modeling. This curve is formed by plotting two statistical indices, namely "sensitivity" or true positive rate and "1-specificity" or false positive rate, on the y-axis and x-axis, respectively^62^. Predictions of the occurrence or non-occurrence of an event can be quantitatively assessed using AUC. AUC is a useful accuracy statistic for vulnerability analysis^63^. The previously unused 30% testing data were processed to form the model used to evaluate its capability. The performance of the generated model improves with increasing AUC values. The following categories of AUC reflect model performance: excellent (0.8–1), good (0.7–0.8), fair (0.6–0.7), and poor (0.5–0.6).

3 $${{\text{r}}}_{{\text{p}}}=\frac{\sum_{{\text{i}}=1}^{{\text{n}}}({{\text{x}}}_{{\text{i}}}-\overline{{\text{x}} })({{\text{y}}}_{{\text{i}}}-\overline{{\text{y}} })}{{\left[\sum_{{\text{i}}=1}^{{\text{n}}}{\left({{\text{x}}}_{{\text{i}}}-\overline{{\text{x}} }\right)}^{2}\sum_{{\text{i}}=1}^{{\text{n}}}{\left({{\text{y}}}_{{\text{i}}}-\overline{{\text{y}} }\right)}^{2}\right]}^\frac{1}{2}},$$

4 $${\text{accuracy}}=\frac{{\text{TP}}+{\text{TN}}}{{\text{TP}}+{\text{TN}}+{\text{FP}}+{\text{FN}}},$$

5 $$\widehat{{\text{K}}}=\frac{{{\text{p}}}_{0}-{{\text{p}}}_{{\text{e}}}}{1-{{\text{p}}}_{{\text{e}}}},$$

6 $${\text{AUC}}= {\int }_{0}^{1}{\text{ROC}}\left({\text{t}}\right){\text{dt}}.$$

### Urban sprawl pressure development

The priority index for handling hazardous urban sprawl in the Bandung metropolitan area is derived from integrating data on the distribution of human economic activity values, environmental degradation values, and potential multi-hazard pressure values in urban areas. The integration process commences by analyzing human economic activity distribution data, which is subsequently combined with reviews of environmental conditions and multi-hazard susceptibility. The resampling method is applied to merge data from various sources that possess distinct resolution characteristics. Resampling plays a vital role in this process by ensuring that image resolutions are properly aligned. By adopting a 30-m resolution as a standard or using the best resolution from the available data, the resolution consistency of the integrated data can be ensured. Pixel transformation to a uniform resolution is a critical step in ensuring the accuracy and relevance of the generated data. Therefore, the resampling method ensures the preservation of data integrity, enabling the generation of a priority index for city expansion with optimal accuracy.

7 $$\mathrm{Conservation \,\,priority}=\sum_{{\text{J}}=1}^{{\text{J}}}(\mathrm{\alpha }\times {\text{HSI}})+(\upbeta \times {\text{LPI}}),$$

## Results

### Human economic activity in urban areas

**Figure 3:**
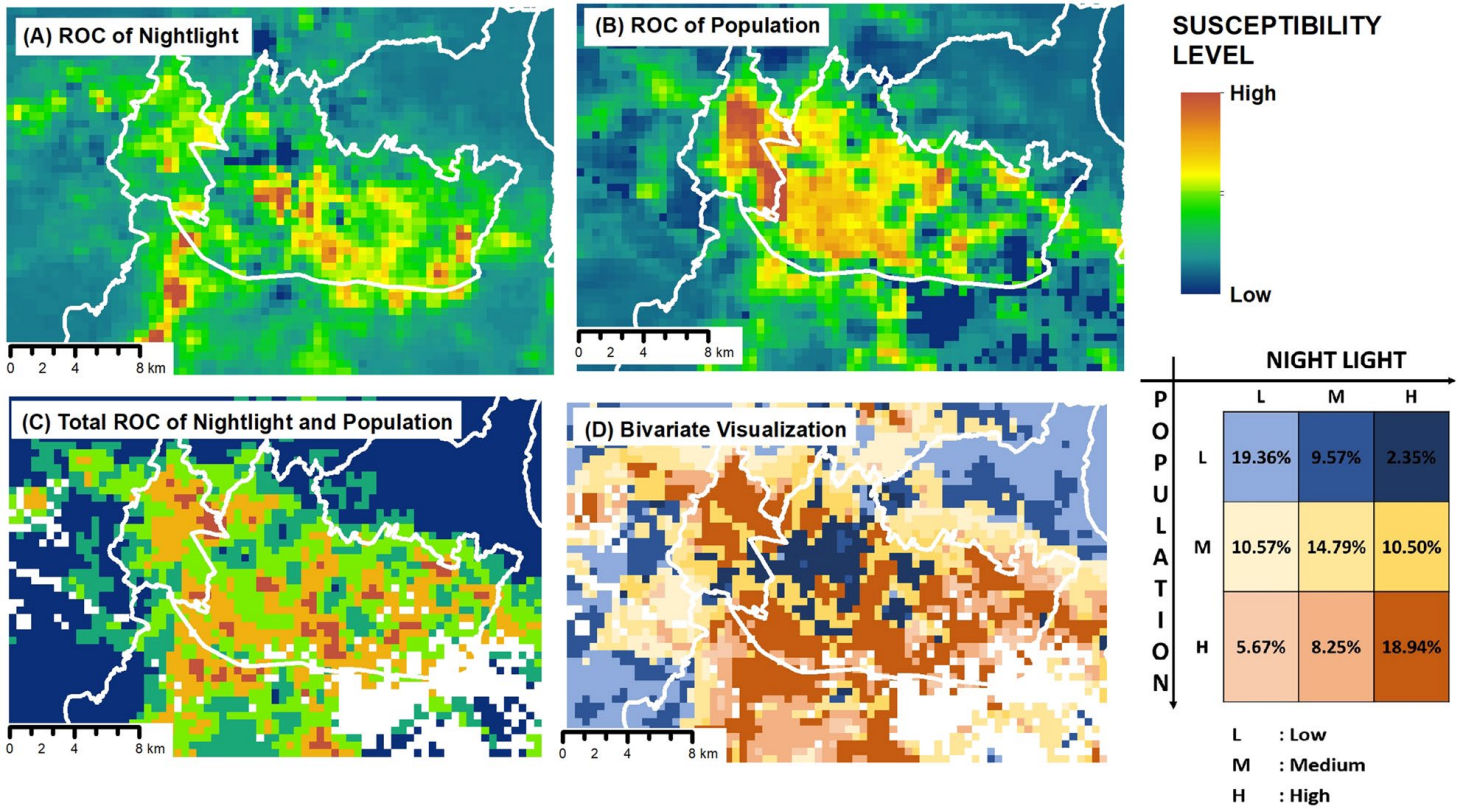
Human economic activity values for urban areas: (**A**) ROC value for the night light parameter, (**B**) ROC value for the population density parameter, (**C**) total ROC value for the combined night light and population density parameters, and (**D**) bivariate analysis result of the ROC for the night light and population density parameters.

In this study, we developed a model for human economic activity in the study area by analyzing the ROC coefficients for long-term population and nighttime light data. Figure 3 depicts the analysis results of the changes in human economic activity. Figure 3A shows the ROC values for nighttime light; Fig. 3B displays the ROC values for population density; and Fig. 3C illustrates the combined total of the change values for population and nighttime light. Additionally, a bivariate analysis was conducted between the changes in population and nighttime light, as depicted in Fig. 3D. In the Bandung metropolitan area, the central part of Bandung City had the highest positive relative ROC value for nighttime light, followed by the southeastern and southern regions of the city. This indicated the emergence of new economic centers in these regions, which increased nighttime light. Moreover, Cimahi City (located west of Bandung City) exhibited the highest positive relative ROC value for population density. This was because of the limited availability of residential land in Bandung City, which had resulted in population growth spreading to surrounding areas. When analyzing the combined effects of nighttime light and population density, the areas surrounding Bandung City showed high total values. This indicated economic and demographic growth in the surrounding regions, stimulated by the expansion of activities from Bandung City to its neighboring areas. The bivariate analysis revealed that 18.94% of the study area comprised areas with both high population growth and high nighttime light. In contrast, the central region of Bandung City, which comprised 19.36% of the study area, experienced minimal changes in nighttime light and population. This could be attributed to the central area having reached its maximum capacity, resulting in no substantial further increases.

### Environmental degradation model

**Figure 4:**
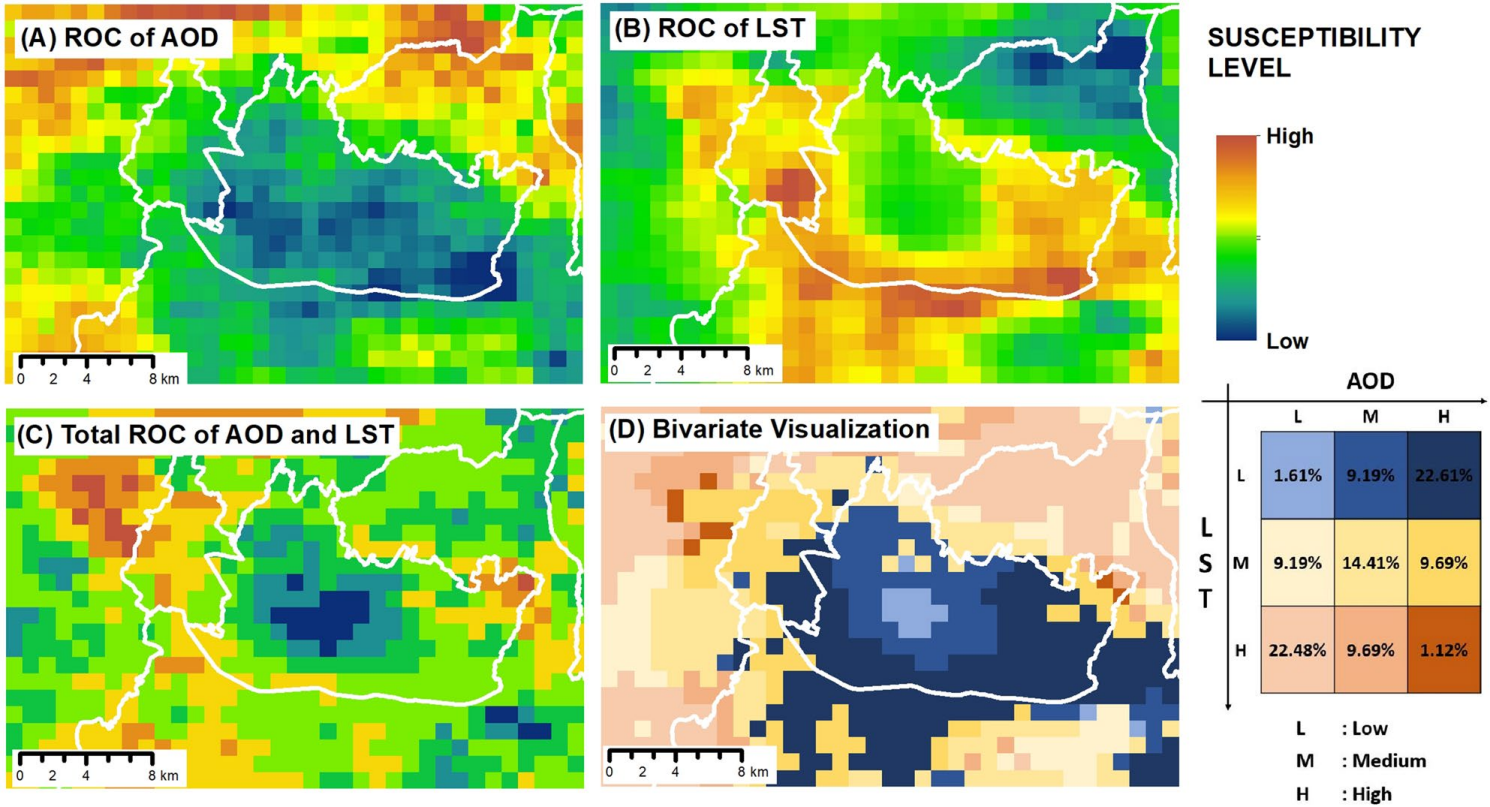
Urban area environmental degradation values: (**A**) ROC value for the aerosol optical depth (AOD) parameter, (**B**) ROC value for the land surface temperature (LST) parameter, (**C**) total ROC value for the combined AOD and LST parameters, and (**D**) bivariate analysis result of the ROC for the AOD and LST parameters.

The ROC results of the environmental degradation pressure modeling, specifically for air pollution represented by AOD data and temperature represented by land surface temperature (LST) data, are presented in Fig. 4. Figure 4A depicts the ROC values for the AOD parameter, indicating higher increases in AOD values in the northern region than those in the southern area. This could be attributed to the construction of new roads in the northern region over the past few years. Figure 4B displays the ROC values for LST. The region with the highest relative ROC values for LST was the southern part of the study area. This pattern aligned with the administrative boundaries of Bandung City and residential areas. Figure 4C exhibits the total ROC values for AOD and LST, with a substantial portion of the study area displaying moderate to high ROC values for both AOD and LST. Figure 4D shows the bivariate visualization of AOD and LST, where the analysis indicates that the northwestern area (Bandung Barat Regency, including the highway) falls into the category of very high positive AOD and LST changes. Based on the bivariate visualization, the class with the highest percentage of area corresponded to the class with a high AOD increase and a low LST, accounting for 22.61% of the total area, followed by the class with a high LST increase and a low AOD, comprising 22.48% of the total area.

### Multi-hazard susceptibility model

**Figure 5:**
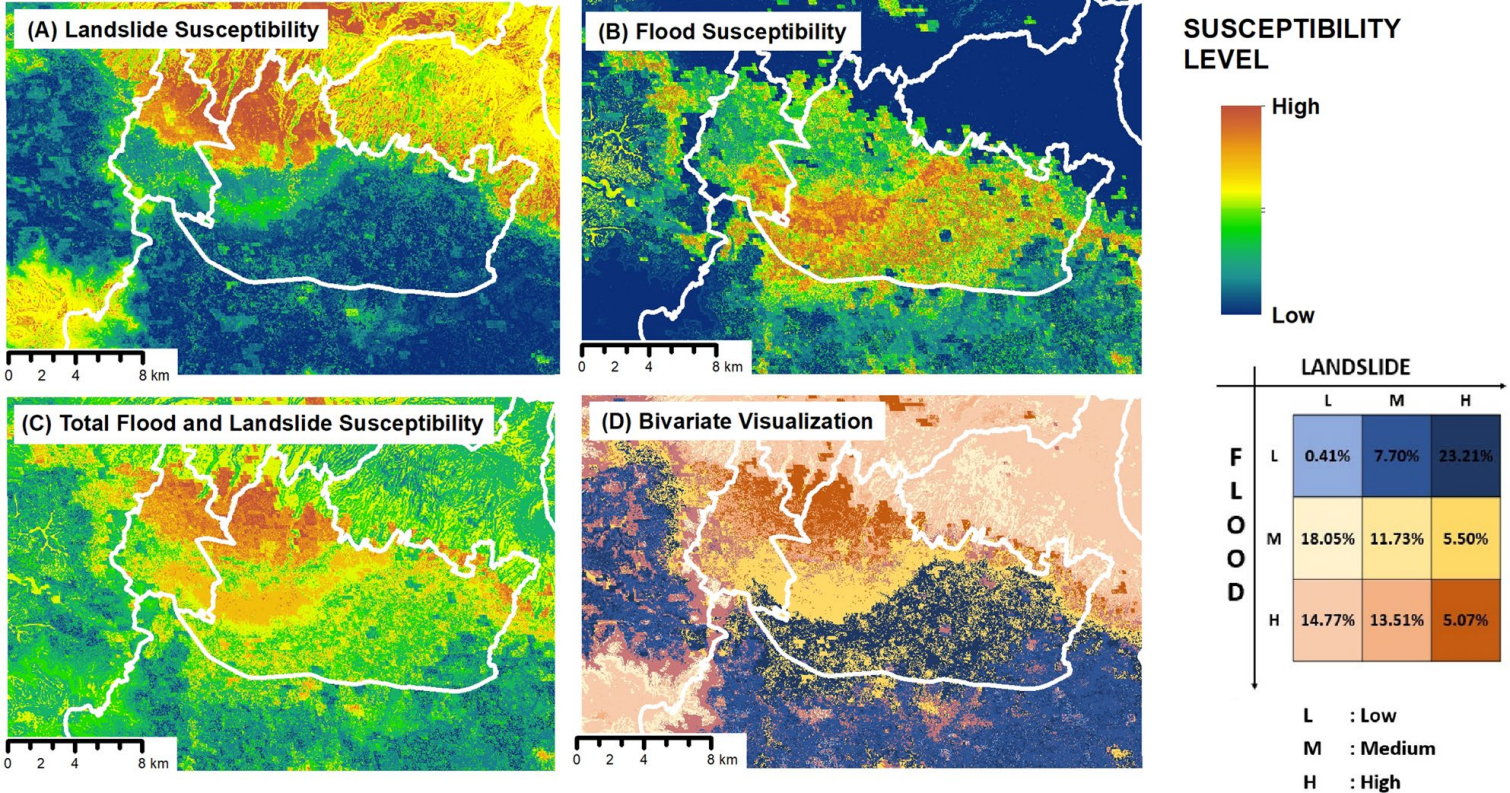
Potential multi-hazard pressure values in urban areas: (**A**) vulnerability index value for the landslide parameter, (**B**) vulnerability index value for the flood parameter, (**C**) total combined multi-hazard value for landslides and floods, and (**D**) bivariate analysis result of vulnerability for landslides and floods.

This study investigated the influence of multi-hazard potential on the rate of hazardous urban sprawl. The multi-hazard vulnerability was obtained by combining the flood and landslide hazard models using RF machine learning (Fig. 5). Figure 5A illustrates the landslide hazard vulnerability index, which indicates that high vulnerability is concentrated in the northern part of Bandung and Sumedang Regency, primarily in forested areas. Figure 5B shows the flood hazard vulnerability index, with vulnerability distributed in the southern part of Bandung and Bandung Regency. This was owing to the presence of two major rivers, namely the Cikapundung River and its tributaries, which generally flowed southward and converged at the Citarum River. Figure 5C displays the combined vulnerability of flood and landslide hazards, with vulnerability levels ranging from moderate to high. Figure 5D depicts the bivariate analysis of flood and landslide vulnerability, which reveals that the Bandung Regency and surrounding areas of Cimahi City have the highest vulnerability values. The percentage of the area with high flood and landslide vulnerability was distributed over 5.07% of the entire study area. The highest percentage area was dominated by low flood and high landslide vulnerability, accounting for 23.21% of the total area, followed by moderate flood and low landslide vulnerability, accounting for 18.05% of the total area.

**Figure 6:**
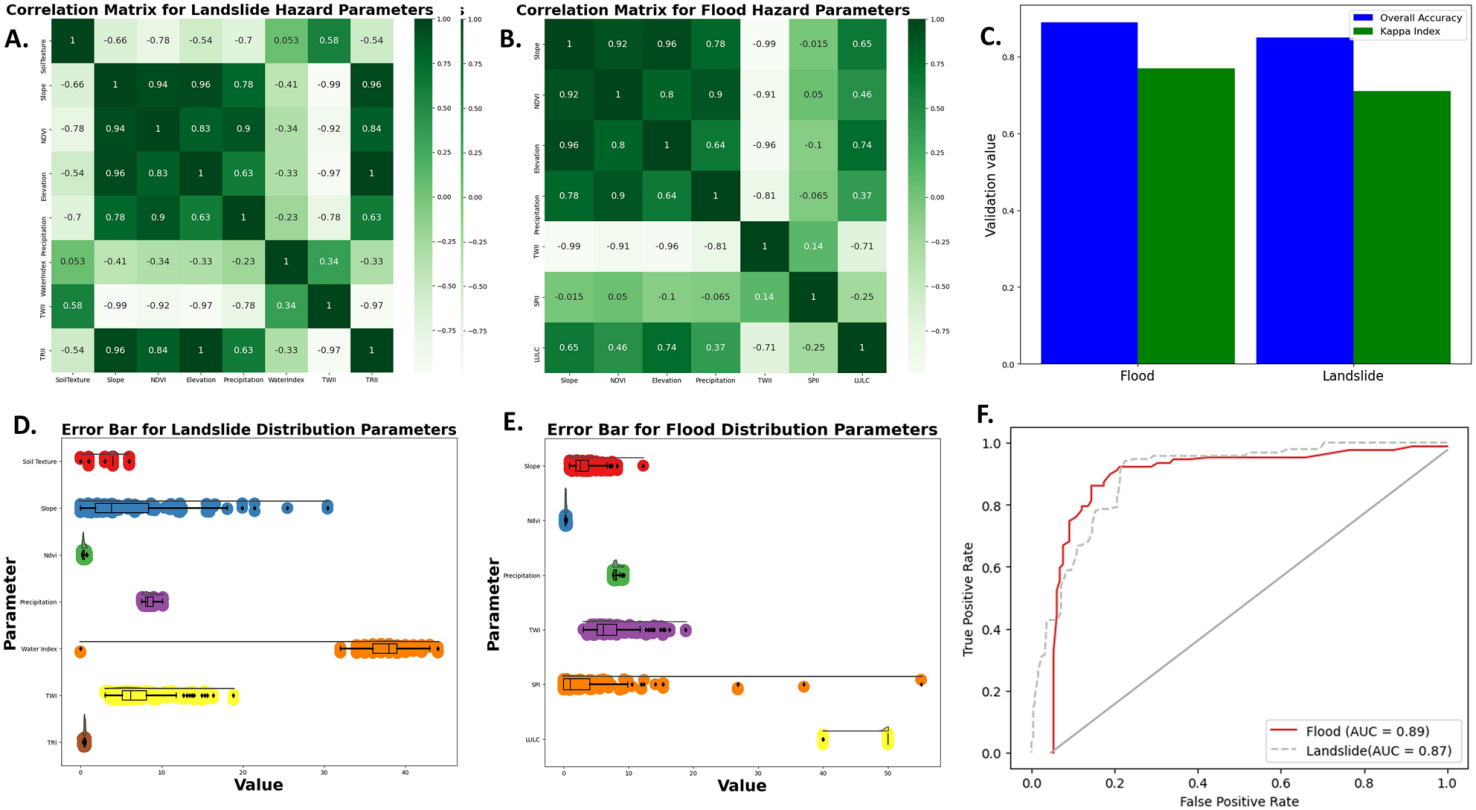
Machine learning statistical analysis for hazard modeling: (**A**) correlation matrix for flood hazards, (**B**) correlation matrix for landslide hazards, (**C)** values of overall accuracy and kappa index for floods and landslides, (**D**) error bars for flood distribution parameters, (**E**) error bars for landslide distribution parameters, and (**F**) AUC values for floods and landslides.

The correlation matrix of parameters was used to analyze the relationships among interrelated parameters in order to develop a hazard model. Figures 6A and B depict the correlation values visualized using a white-to-dark-green color scale, representing values from − 1 to 1 for both floods and landslides, respectively. A value of 1, denoted by the dark green color, indicates a strong correlation between parameters. This strong correlation suggests that similar parameters have a dual impact on the vulnerability model. In the case of flood and landslide hazards, slope and elevation have a correlation close to 1. This suggests that slope and elevation have a nearly identical contribution to the flood vulnerability model. Figure 6C depicts The overall accuracy, and kappa index values for flood hazards were 0.89, and 0.77, respectively, whereas for landslide hazards, they were 0.85, and 0.71 indicating good model performance. Figures 6D and E depict the distribution of flood and landslide vulnerability parameter values. The flood hazard exhibits dispersed parameter values for slope, topographic wetness index (TWI), and stream power index (SPI) ranging from 0 to 20. Similarly, the parameter values for landslide hazard vulnerability are evenly distributed across slope and TWI parameters. The ROC curve assesses the true positive (TP) and true negative (TN) indices, which represent correctly classified hazard and non-hazard samples, respectively. Conversely, false positives (FPs) and false negatives (FNs) indicate hazard and non-hazard samples that were misclassified, respectively (Fig. 6F). The TP, TN, FP, and FN indices are graphically employed to evaluate the predictive capability of the model, where a higher proportion of TP and TN signifies a superior model. Figure 6F depicts the ROC curve and the AUC values of the flood and landslide hazard models developed using the RF machine learning algorithm. The AUC values for flood hazards were 0.89, whereas for landslide hazards were 0.87, indicating good model performance.

### Integrated hazardous urban sprawl index

**Figure 7:**
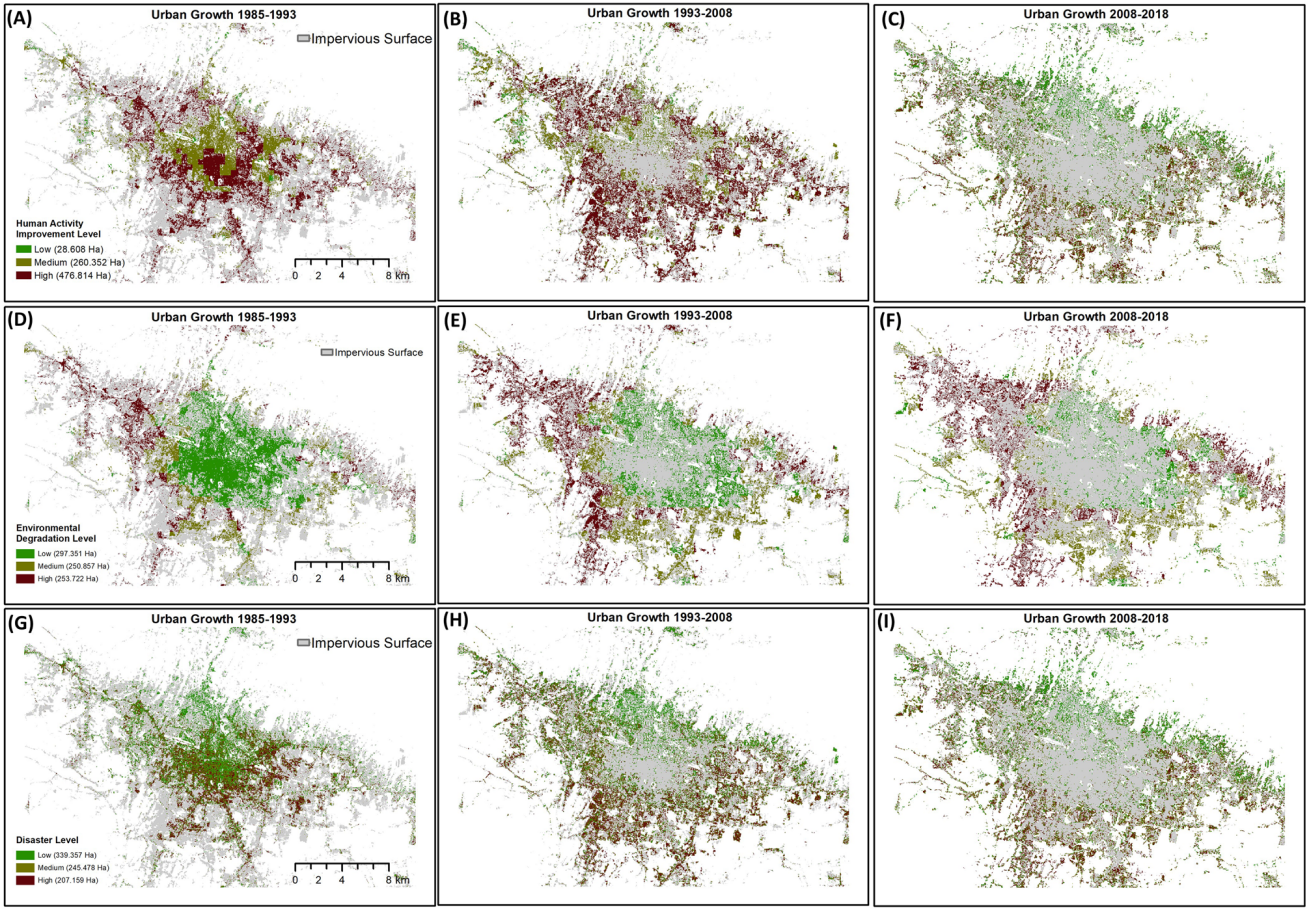
Hazardous urban sprawl index products that are based on three time periods of urban movement in relation to human economic activity, environmental degradation, and multi-hazard.

In the analysis of identifying the hazardous urban sprawl index, urban movement was divided into three time periods: 1985 to 1993, 1993 to 2008, and 2008 to 2018. There were three categories of hazards: human economic activity, environmental degradation, and multi-hazard. These data were integrated to generate nine analyses. Figure 7 illustrates the levels of human economic activity, environmental degradation, and multi-hazard for each group of urban movement time periods. Generally, areas within the groups of urban movement time periods that exhibit higher levels of environmental degradation, human economic activity, and multi-hazards should be prioritized to address urban sprawl issues. Figures 7A–C depict the levels of human economic activity for the three groups: 1985–1993, 1993–2008, and 2008–2018, respectively. The 1993–2008 group showed the largest area with high levels of human economic activity, covering an area of 172,776 ha. However, the growth of human economic activity in the 2008–2018 group appeared to have decreased by approximately 13.17%. Figures 7D,F 6E show the environmental degradation levels for the three groups of urban growth time periods. Generally, there was an increase in the ROC values in the high class for environmental degradation factors each year. The highest increase in the high class occurred during the urban growth period from 1985 to 1993, covering an area of 52,262 ha. The 1993–2008 group experienced a considerable increase of approximately 71.11%, covering an area of 91,134 ha. Lastly, the hazard level is indicated in Figs. 7G–I, with the highest area of ROC in the high class occurring in the 1993–2008 group, increasing from 66,393 ha to 82,083 ha and then decreasing in the 2008–2018 group to 58,563 ha.

**Figure 8:**
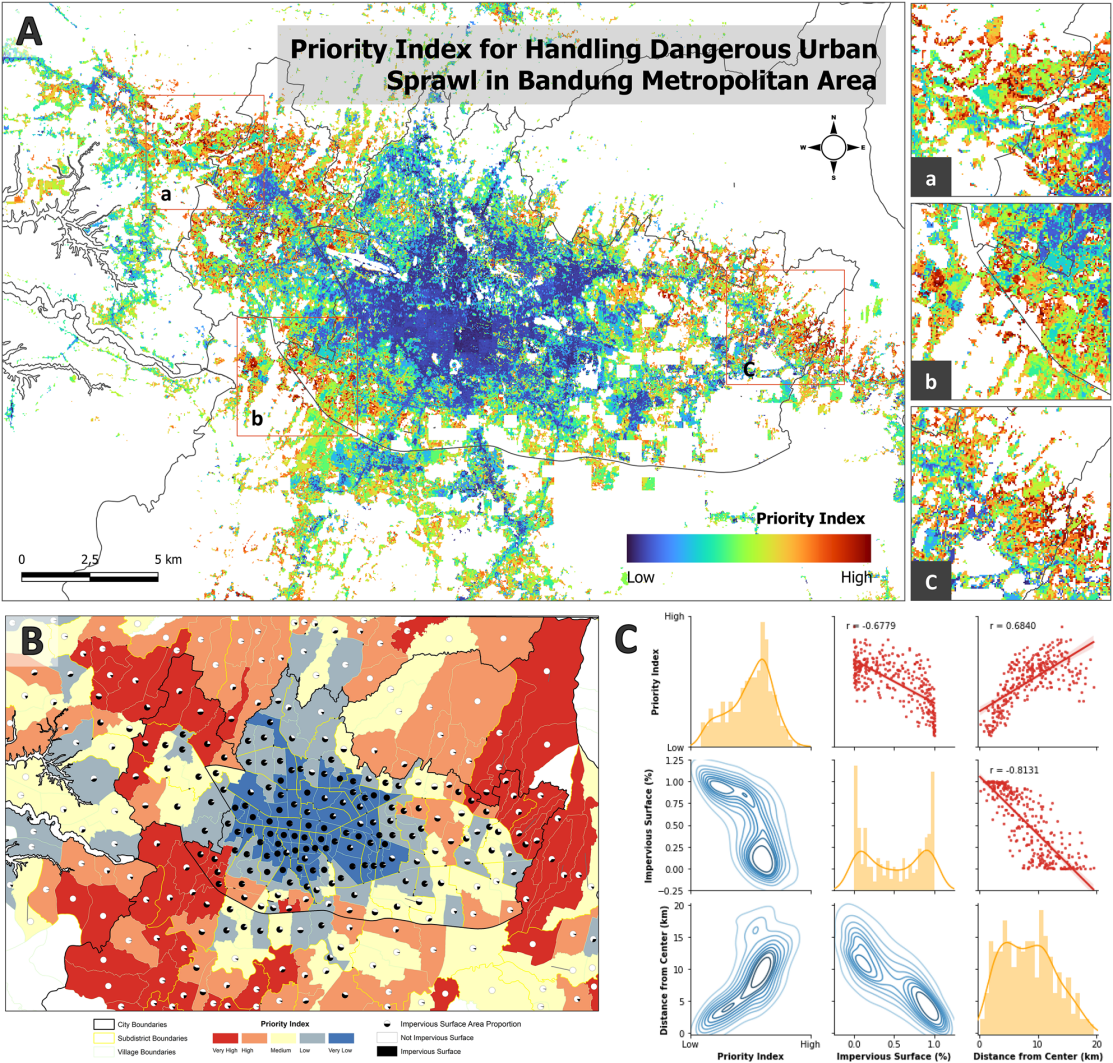
(**A**) Priority index product for managing hazardous urban sprawl, (**B**) priority index for managing sub-district urban sprawl, and (**C**) pair grid graph depicting the relationship between the priority index, impervious surface, and distance from the center.

A combined model in the form of a priority index for addressing hazardous urban sprawl was formed through the integration of human economic activity, environmental degradation, and multi-hazard parameters, visualized at levels from high to low (Fig. 8A). The low class is represented by a dark blue color, whereas the high class is depicted by a dark red color. A low-priority index denotes low levels of human economic activity, environmental degradation, and hazards. Conversely, a high-priority index indicates high levels of human economic activity, environmental degradation, and hazard. The higher the index value in an area, the more urgent the need for intervention (such as restrictions or prevention) to address hazardous urban sprawl in that area. Figure 8A shows that the dominant high-priority index is dispersed on the outskirts of urban areas, particularly around the zoomed-in areas A, B, and C. Area A corresponds to West Bandung Regency and Cimahi City; area B includes Bandung Regency, Bandung City, and Cimahi City; and area C comprises Bandung City and the eastern part of Bandung Regency. Regions with high-priority index values require more government attention to promptly address the negative impacts of hazardous urban sprawl.

The priority index for addressing hazardous urban sprawl was analyzed at the neighborhood level in the Bandung metropolitan area (Fig. 8B). Based on the average priority index values in each neighborhood, natural breaks were used to divide the classes into five priorities: very low, low, moderate, high, and very high. The priority index values ranged from very low to very high. The management of the urban sprawl rate at the neighborhood level was compared with the proportion of impervious surface area versus total area through pie chart visualization. The larger the size of the black slice on the pie chart, the higher the proportion of impervious surface area in that neighborhood. In Fig. 8B, neighborhoods located in the central area of Bandung City predominantly exhibit a high proportion of impervious surface area but a low urban management priority index. Conversely, neighborhoods on the outskirts of the Bandung metropolitan area, including parts of Cimahi, West Bandung Regency, and Bandung Regency, had low percentages of impervious surface area but a high-priority index for urban management.

The relationships among the variables of priority index (low-to-high scale), impervious surface (%), and distance of neighborhoods from the center of the Bandung metropolitan area (km) are illustrated in Fig. 8C. These relationships were visualized via a histogram, kernel density, and scatter plot. Based on the graph, there was a negative correlation (r = − 0.6779) between the priority index and impervious surface, with neighborhoods with high impervious surface coverage (approaching 100%) falling into the very low-priority class and neighborhoods with low impervious surface coverage (approaching 0%) exhibiting medium to high-priority index values. In contrast, the graph depicting the relationship between the priority index and distance from the center of the Bandung metropolitan area showed a positive correlation of 0.6840. The central areas of Bandung City had low-priority index values, whereas the neighborhoods on the outskirts of the city had high-priority index values. This phenomenon indicates population growth on the outskirts of the Bandung metropolitan area, such as in parts of the Bandung Regency, West Bandung Regency, and Cimahi City. However, these inhabited areas pose a high risk in terms of both natural hazards and environmental quality. Efforts should be made to limit urbanization in these areas and maximize land use for other sectors.

## Discussion

### Comparing residential density and the hazardous urban index

**Figure 9:**
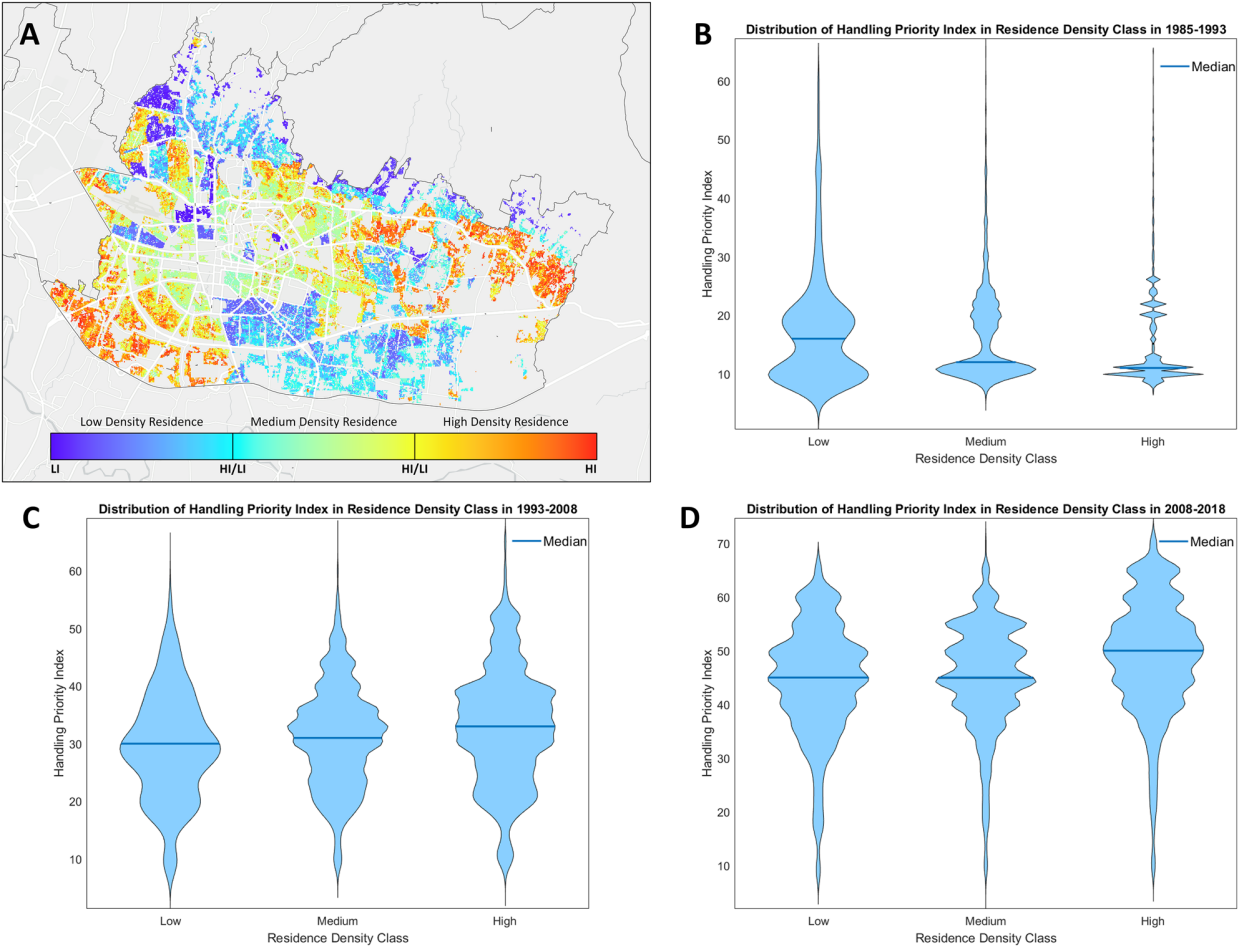
(**A**) Distribution map of residential density classes and the handling priority index, (**B**) distribution of the handling priority index in residence density classes from 1985 to 1993, and the distribution of the handling priority index in residence density classes for different time periods: (**B**) 1985–1993, (**C**) 1993–2008, and (**D**) 2008–2018.

In this study, the handling priority results are compared with the spatial planning data on residential density from the government of Bandung^47^. The purpose of this comparison is to evaluate spatial planning based on the developed handling priority index. Figure 9 shows the comparison between government spatial planning and the handling priority index. Figure 9A illustrates the distribution between residential density classes and the handling priority index. In the northern part of Bandung, there is a low residential density, whereas some areas exhibit a high handling priority index. This is attributed to the region being a highland area with numerous plantations, coupled with its reputation for natural beauty and tourist attractions, making it an appealing residential location. The likelihood of urban sprawl in this area is increasing because of the pleasant environment and increasing economic activities spurred by growing tourist destinations. This analysis is conducted to observe the distribution patterns of building classes and the handling priority index (HPI) across different years. The results reveal an annual increase in the HPI for each class, indicating the progression of urban sprawl in Bandung throughout the years. Figure 9B highlights that older buildings with low residential densities tend to have the highest HPI. Conversely, for high-density classes, the highest index differs in Fig. 9C and D. This implies that older buildings currently serve as focal points for dense population activities, acting as pioneers for future occupants, thereby contributing to the observed increase in urban sprawl.

### Trend analysis of a highly hazardous urban sprawl area

**Figure 10:**
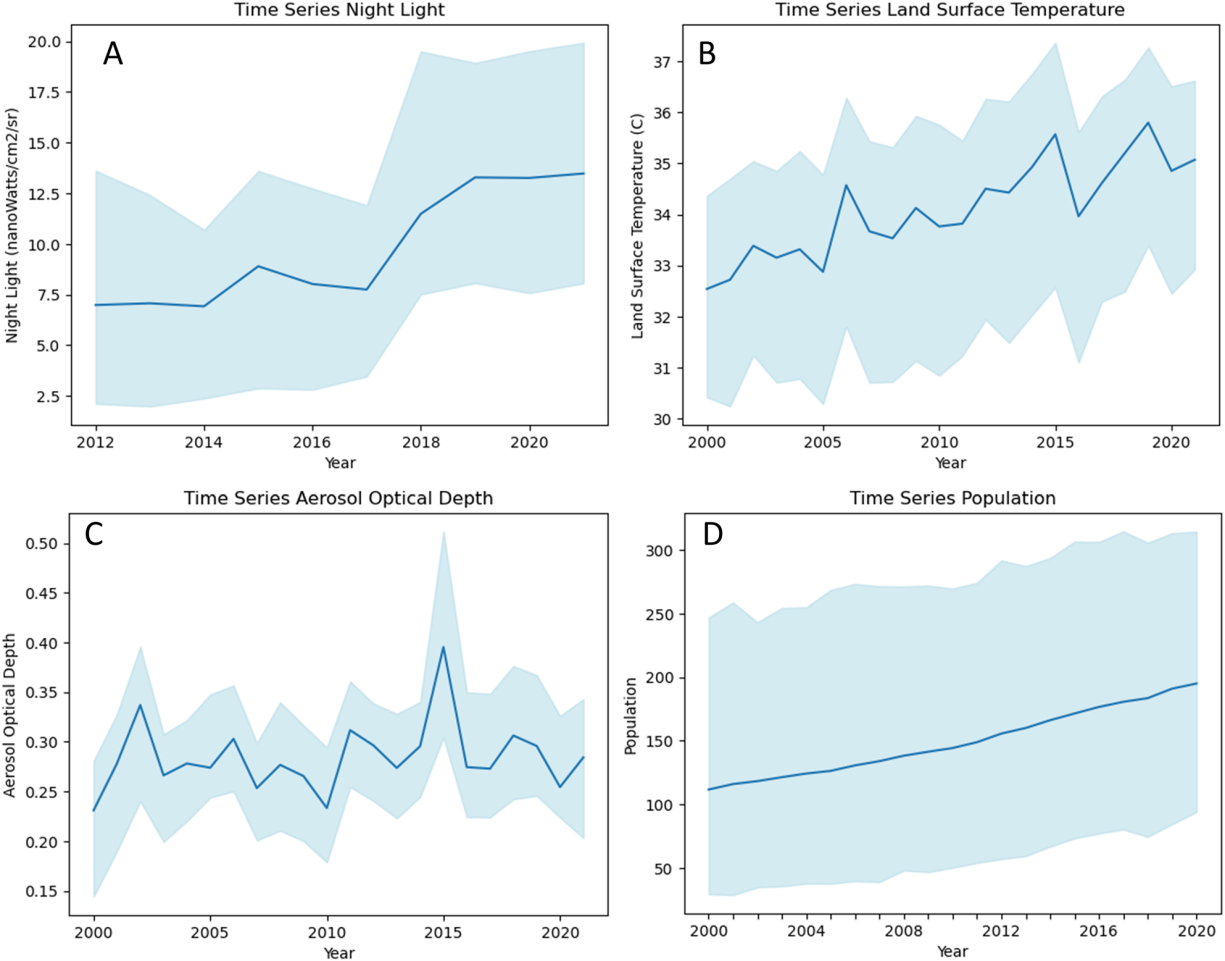
Time series distributions of the four indicators: (**A**) nighttime lights, (**B**) surface temperatures, (**C**) aerosol optical depth (AOD), and (**D**) population, for all grid pixels of impervious surfaces within sub-districts categorized as highly prioritized for managing hazardous urban sprawl.

Time series analysis was performed on neighborhoods with a very high priority index for addressing urban hazards to identify changes in environmental and socio-economic factors over time. Figure 10 presents the extracted values of impervious surface from the entire pixel grid for the parameters of nighttime light, surface temperature, AOD, and population. The time series graph displays the maximum, minimum, and average values of all analyzed grid pixels. In Fig. 10A, it can be observed that neighborhoods with a very high priority class experienced an increase in nighttime lights from 2012 to 2021. This indicates that areas affected by hazardous urban sprawl are experiencing a rise in economic activity, as evidenced by the increase in nighttime lighting. Figure 10B–D indicate that neighborhoods in the high-priority class also experience significant increases in temperature, AOD, and population. This highlights the interdependence and mutual impact of these four indicators. Population growth and an increase in nighttime lights had reciprocal effects, which in turn impacted the temperature and AOD trends. However, the increase in AOD was not as pronounced, most likely as a result of the rapid movement of AOD in the atmosphere caused by factors such as wind. Additionally, the limited research area might not have captured the full extent of atmospheric movements that influence AOD.

### Limitations and future study directions

This study had several limitations. One limitation was the presence of data with different time ranges, which was a result of the availability of observational data. The difference in observation years can affect the starting point for detecting changes in the study area, potentially leading to distinct patterns that allow for changes to occur. Additionally, linear analysis was used to identify increases, assuming that annual changes were constant. However, in some cases, non-linear changes are possible. The approach for each parameter model used in this study could be enhanced by considering additional factors that influence the outcomes. The human economic activity factor could be improved by incorporating important economic activity points that may accelerate urban sprawl^64^. The environmental degradation factor could be enhanced by considering air quality observation factors, which can indicate increased human economic activity in the area. The hazard factor could be improved by considering other hazards, such as earthquakes and land subsidence^65,66^. The determination of multi-hazards could be enhanced by using multi-machine learning to identify locations with high hazards. The analytical data provided could be improved by analyzing individual buildings to identify those in need of further intervention. This would provide insight into buildings that require specific attention. This study mainly focused on the horizontal aspect of urban sprawl, but it could be expanded to investigate urban development from a vertical perspective by incorporating changes in building height^67^. The methods used in this study could be applied to address other issues, such as urban planning in response to overpopulation^68–70^.

## Conclusions

The management of hazardous urban sprawl is a long-term process that requires sustainable efforts, coordination, and adaptive strategies. With the development of a prioritization index model for managing hazardous urban sprawl, it is anticipated that urban expansion can be addressed while simultaneously promoting sustainable and livable communities. This study conducted three assessments to determine priority areas for intervention: human economic activity, environmental degradation, and multi-hazard. In terms of human economic activity, the central area of Bandung City showed high levels of nighttime lights, whereas the western area (Cimahi) experienced substantial population growth. Areas with low levels of nighttime lights and population accounted for 19.36% of the total study area. In terms of environmental degradation, the northern region exhibited a higher AOD compared to that of the southern region. The highest increase in LST was found in the southern part of the study area. In the research area, regions with high AOD and low LST values dominated, accounting for 22.61% of the total area. In terms of potential hazards, the northern region posed a higher risk of landslides owing to its highland nature, whereas the southern region showed a high risk of flooding. Based on the integration of multiple hazards, the largest area was classified as having a low flood risk but a high landslide risk, covering 23.21% of the total area. The analysis of hazardous urban sprawl over three different time periods (1985–1993, 1993–2008, and 2008–2018) revealed that the 1993–2008 period had the highest increase in human economic activity, reaching 172,776 ha. The 1985–1993 period experienced the highest level of environmental degradation in the study area. Meanwhile, the 1993–2008 period showed the highest concentration of multi-hazard locations. The combined model of hazardous urban sprawl, incorporating the three parameters, indicated that the highest priority for intervention was on the outskirts of urban areas, specifically in West Bandung Regency, Cimahi, Bandung Regency, and East Bandung Regency. Regions with high-priority indices require greater attention from the government to mitigate the negative impacts of hazardous urban sprawl.

## Data Availability

The datasets generated during and/or analysed during the current study are available from the corresponding author on reasonable request [AD: albert@itb.ac.id].
